# Intestinal microbiota research from a global perspective

**DOI:** 10.1093/gastro/goac010

**Published:** 2022-04-11

**Authors:** Jordyn T Wallenborn, Pascale Vonaesch

**Affiliations:** 1 Department of Epidemiology and Public Health, Swiss Tropical and Public Health Institute, Basel, Switzerland; 2 University of Basel, Basel, Switzerland; 3 Department of Fundamental Microbiology, University of Lausanne, Bâtiment Biophore Campus UNIL-Sorge, Lausanne, Switzerland

**Keywords:** intestinal microbiota studies, geographic influence, environment, societal impact, global health, gut microbiome

## Abstract

The intestinal microbiota plays a crucial role in health and changes in its composition are linked with major global human diseases. Fully understanding what shapes the human intestinal microbiota composition and knowing ways of modulating the composition are critical for promotion of life-course health, combating diseases, and reducing global health disparities. We aim to provide a foundation for understanding what shapes the human intestinal microbiota on an individual and global scale, and how interventions could utilize this information to promote life-course health and reduce global health disparities. We briefly review experiences within the first 1,000 days of life and how long-term exposures to environmental elements or geographic specific cultures have lasting impacts on the intestinal microbiota. We also discuss major public health threats linked to the intestinal microbiota, including antimicrobial resistance and disappearing microbial diversity due to globalization. In order to promote global health, we argue that the interplay of the larger ecosystem with intestinal microbiota research should be utilized for future research and urge for global efforts to conserve microbial diversity.

## Introduction

The microbiota generally includes a community of bacteria, archeae, fungi, protozoa, worms, and viruses that live inside and on the human body as well as all the genes that they jointly encode [1]. Playing a crucial role in our health over the life course [2], the microbiota is generally believed to be inherited at birth from the mother, maturing during the first months of life [3]. At around two or three years of age, the intestinal microbiota reaches an adult-like composition and complexity [3] and remains relatively unchanged until senescence, making the first years of life critical for optimal microbial colonization [4].

In predicting life-course trajectories of health, deviations of bacterial communities from a healthy state (i.e. dysbiosis) of the infant intestinal microbiota have been associated with a variety of morbidities [5] and several infectious and chronic diseases, including necrotizing enterocolitis, inflammatory bowel diseases, malnutrition, metabolic conditions (e.g. obesity), and atopic diseases including allergies and asthma [6]. Alpha diversity (i.e. number of distinct members) and beta diversity (i.e. variability of microbial communities) of gut microbial taxa may also play essential roles in child neurodevelopment and optimal growth [7].

As a healthy seed microbiota is inherited from the mother, through skin-to-skin contact, breast milk, and/or the vaginal tract, a mother’s microbial dysbiosis can be passed on to the child [3]. In general, the bacterial species in the intestinal microbiota is highly variable between individuals, but is generally dominated by *Actinobacteria* and *Firmicutes* [8, 9]. However, the intestinal microbiota typically encompasses similar bacterial strains grouped by encoding function, suggesting that the human microbiota is based on functional properties rather than a specific taxonomic assembly [10, 11].

Understanding the complex assembly of an individual’s gut microbial community is of great interest for immunology, microbiology, and—more recently—public health interventions [12]. In fact, personalized medicine—a novel and potentially groundbreaking field—may target the intestinal microbiota as a therapeutic solution for various diseases, including some of the main public health concerns of modern times such as ischemic heart disease [13], stroke [14], chronic obstructive pulmonary disease [15], or cancer [16, 17].

Our review provides an overview of individual, environmental, and geographic factors that shape the human intestinal microbiota utilizing a global perspective. We also discuss the importance of the intestinal microbiota for life-course trajectories and global health. Lastly, we review promising interventions that promote a healthy intestinal microbiota and global health.

## Microbiome development on an individual scale

### Effect of mode of delivery on future health

In the immediate period following birth, the infant’s immune system is undeveloped due to the near-sterile environment of the mother’s womb [3, 18–20]. The first stages of immune-system maturation and gut colonization are heavily shaped by the birthing process (i.e. mode of delivery). Exposure to vaginal and fecal microbial communities during natural childbirth is a critical factor in “seeding” an infant’s microbiota composition [4, 21]. Vaginal taxa from the mother have also been found to transiently colonize the child’s fecal and airway microbiota [22]. Vaginal microbiota communities are typically dominated by *Lactobacillus* species [23, 24], specifically *L. iners*, *L. crispatus*, *L. gasseri*, or *L. jensenii*; yet, significant differences are seen between North American women from different ethnic groups (White, Black, Hispanic, and Asian) [25]. When a misbalance in vaginal microbiota occurs, such as a lower abundance of *Lactobacillus*, bacterial vaginosis is likely to occur—resulting in unwanted perinatal outcomes, including preterm birth (e.g. infant born at <37 weeks’ gestation) [26]. Yet, women with a vaginal microbiome dominated by *L.crispatus* seem to have a lower risk of preterm birth [26, 27]. Maternal fecal microbiota can also have profound effects on birth outcomes, including gestational age at birth, birthweight, and neonatal growth [28].

Infants born preterm are exposed to an undeveloped vaginal microbiota, as the vaginal microbiota only increases in its diversity after 36 weeks of gestation [29]. In fact, the maternal vaginal and fecal microbiota changes during pregnancy; therefore, if a child is born at an earlier stage of pregnancy, the child will not be exposed to a mature fecal or vaginal microbiota. In early pregnancy, an initial increase in butyrate-producing strains in the feces was found among 91 pregnant women [30]. Later in pregnancy, there was a significant decrease in alpha diversity and an increase in beta diversity in the fecal microbiota—which was accompanied by an increase in *Enterobacteriaceae* and *Actinobacteria*, and a decrease in *Faecalibacterium* [30, 31]. In late pregnancy, the vaginal microbiota shifts towards a microbiome dominated by *Lactobacillus* and concomitantly a decrease in both alpha and beta diversity [32].

Even among infants born at term (i.e. end of pregnacy), a dysbiotic microbiota from the mother can be passed to her child [33]. In two small studies in Spain [34] (16 cases and 26 controls) and in the USA (77 subjects overall) [35], infants from obese mothers were found to inherit a dysbiotic microbiota. In mothers suffering from intestinal bowel disease, aberrant intestinal microbiota composition was found throughout pregnancy and their children presented with a changed seed microbiota, affecting immune markers when transferred to germ-free mice [36]. Recent evidence also links the maternal third-trimester microbiota to child behavior in their offspring, emphasizing the importance of the inherited seed microbiota on the healthy development of children [37].

Infants born by Cesarean section (C-section) have a disruption to the mother–newborn transmission of microbiota, as they are not exposed to the vaginal and fecal microbiota at birth and only acquire a seed microbiota from the mother’s skin and the environment [3, 38]. Mothers who undergo a C-section are often provided wih intrapartum antibiotics in order to prevent surgical infection [39], which has a deleterious effect on microbiota [40]. As a result, infants born vaginally show higher levels of *Bifidobacterium* and lower levels of *Enterococcus* and *Klebsiella* than infants born by C-section; however, these differences appear regardless of intrapartum antibiotic use [41]. A lack of exposure to these microbial communities may disrupt the normal infant intestinal microbiota development, resulting in an immune system that does not function properly and increases the risk of disease [42]. For example, higher levels of *Bifidobacterium* are consistently found in vaginal-born infants than infants born by C-section [43] and is important for host defense against pathogens [44].

Responding to the major lifelong implications for infant intestinal microbiota development and life-course health among infants born by C-section, medical interventions are utilizing maternal vaginal microbes to artificially inoculate infants by swabbing an infant’s face, nose, and ears with vaginal fluid [45]. However, a pilot study providing oral administration of vaginal microbes to children born by C-section in New Zealand calls into question the importance of vaginal microbes for seeding [46]. There is also an ongoing debate on which maternal microbiota, specifically vaginal or fecal, is more important for initial seeding of the child microbiota and whether transferring maternal vaginal or fecal microbiota to infants born by C-section can restore the disturbed seed microbiota [47]. Two recent studies demonstrated that strains from different maternal microbiota are transmitted to the child; however, most of the maternal strains found in the infant’s intestinal microbiota come from the maternal intestinal microbiota—which leads to a more stable colonization than strains from other sources, such as the vaginal microbiota [33, 48]. A recent pilot study corroborated this finding, showing that fecal microbiota transfer from the mother corrects disturbances in early-life microbiota among infants born by C-section [49]. Further, fecal microbiota of the infant is more similar to maternal fecal than vaginal microbiota [50]. All of the aforementioned studies were limited by participation size; therefore, further research is needed to reproduce these scientific results and assess the potential use of microbiota restoration interventions. Due to the global increase and trend of planned C-sections, identification of a microbiota restoration strategy is of the utmost importance for global health.

Therefore, in terms of global health, a healthy maternal microbiota passed on to the child through a natural birth process sets the stage for a healthy seed microbiota in newborn infants and a positive effect of lifelong health.

### Influence of breastmilk bioactives and microbiota on the intestinal microbiota of the child and future health

Historically, breast milk was considered sterile; however, accumulating evidence using culture-dependent and sequencing technologies shows the presence of a specific breastmilk microbiota, dominated by *Staphylococci*, *Streptococci*, lactic acid bacteria, and *Bifidobacteria* [51–53]. The human microbiota is especially rich in human milk oligosaccharides (HMOs), which are a potent prebiotic for the developing infant’s gut microbiota. Breast milk also contains a plethora of bioactive compounds including immune cells, immunoglobulins, antimicrobial peptides, fatty acids, polyamines, and oligosaccharides [54, 55]. Currently, breastmilk microbiota is recognized as the second step in seeding the infant gut, with ∼25% of breastmilk microbiota being transferred to the infant’s intestinal microbiota [56], including gut-associated anaerobes [51, 57].

The specific microbiota and bioactive components in breast milk also influence the life-course trajectory of the infant’s intestinal microbiota community. Both breastmilk microbes and its bioactive substances play a direct role on the developing intestinal microbiota, contributing to a decreased risk of asthma and allergy in later life [58]. Immune-modulating compounds found in breast milk also help reduce the likelihood of infections [59]. Further, infant ingestion of breastmilk taxa as well as the prebiotic effects of breastmilk components may promote immune programming [54, 58, 60]. Thus, breast milk provides two critical components for infants: (i) a source of new bacterial species that shapes the intestinal microbial community assembly and (ii) specific HMOs and other bioactives that help create a sustained colonization of the right strains in the developing fecal microbiota of the infant.

Breastfeeding duration also heavily influences the intestinal microbiota. A meta-analysis of seven studies that included exclusive breastfeeding information and infant intestinal microbiota found immediate and consistent differences in the intestinal microbiota between exclusively breastfed and non-exclusively breastfed infants—persisting long after 6 months of age [61]. The importance of exclusive breastfeeding crosses geographic boundaries, as the seven studies were conducted across different populations [61]. A large Canadian cohort reported that infant intestinal dysbiosis resulting from intrapartum antibiotics was improved by exclusive breastfeeding and a longer breastfeeding duration [40]. A higher microbial diversity was also reported in exclusively breastfed infants than in non-exclusive breastfed infants at 6 and 14 weeks postpartum; however, a longer follow-up period is needed to ensure permanent and long-term benefits [62].

While still under investigation, breastfeeding—either through breastmilk microbiota or the immunomodulatory and prebiotic substances—plays a crucial role in the initiation and maintenance of a healthy intestinal microbiota. Breast milk may provide a viable strategy for promoting lifelong health by optimizing or correcting gut microbial dysbiosis. One prime example is the difference between breastfeeding and formula feeding in preterm infants.

Preliminary evidence from an observational cohort study (*n* = 69) suggests that human-donor breastmilk-fed preterm infants have gut microbial profiles that closely resemble mothers’ own milk-fed preterm infants, whereas formula-fed infants had significantly less microbial abundance [63]. Hence, it is possible that providing donor human milk to infants not receiving breast milk could support gut microbial development and modulate gut dysbiosis [62].

Overall, breast milk seems to be a major contributor to proper microbiota development and lifelong health.

### Effect of early-life antibiotics on future health

In the last 70 years, most communities have seen a consistent increase in the use of antibiotics, often at a very early age. Antibiotics significantly disrupt the intestinal microbiota and have long-term implications for life-course health. Antibiotic use has been shown to reduce the diversity of gut microbial communities and increases the likelihood of antibiotic-resistant organisms [64]. Disruption to the intestinal microbiota in early life by antibiotics may cause irreversible damage, as microbial communities often fail to completely return to the pre-antibiotic state [65]. Destruction to the intestinal microbiota caused by early-life antibiotic use is likely the causal link between antibiotic use and poor health outcomes [66]. Experimental research from mice receiving fecal microbiota transplant from antibiotic-exposed children showed reduced growth compared with mice receiving a transplant from children not exposed to antibiotics [67].

Research also consistently demonstrates an effect between early-life antibiotics and child growth in humans. Higher body mass indexes (BMIs) were found among boys and girls <6 years of age if they were exposed to antibiotics during the neonatal period [67]. A dose–response relationship was also found between antibiotic use and childhood BMI z-score, showing a higher BMI with a higher number of exposures [68]. Further, boys had significantly smaller height and weight gains if exposed to antibiotics during the neonatal period; however, this association was not found among girls [67]. Interestingly, maternal antibiotic use in pregnancy has also been associated with an increased risk of obesity and asthma in childhood [69–71].

Thus, antibiotics in early life pose a clear risk to a proper microbiota development and can affect lifelong health. They should thus be used very carefully, especially in the first 1,000 days of life.

## Microbiome development on a global/community scale

We briefly reviewed how different exposures [43] in the first 6 months postpartum, including mode of delivery [40, 72–79], gestational age at birth [78], early-life breastfeeding or formula feeding [80], and the use of antibiotics [40], influence intestinal microbiota development. However, additional host and environmental factors later in life also shape the intestinal microbiota. These include diet [81–83], systemic inflammation [84], disease, household and nutritional parameters [85], age, micronutrient deficiencies [86], general health status [87], medical prescriptions [88], genetics [89], and the immune system [87]. Environmental factors are hypothesized to have the biggest effect on the intestinal microbiota [90]. Yet, many studies on the human microbiome are limited by low sample sizes. Technical differences in data generation also make an unbiased meta-analysis implausible. Further, it is difficult to disentangle vertical transmission of the microbiota due to passing of the microbiota and host genetics from mother to child.

Diet is a main environmental factor that differs within and between geographic regions, creating individual and community-level differences in the human microbiota [81, 82]. Short-term consumption of a diet composed entirely of animal-based or plant-based products showed that diet was able to shape the microbial profile more profoundly than inter-individual differences with an increased presence of bile-tolerant microorganisms in the context of an animal-based diet and an increase in the level of polysaccharide-metabolizers in the plant-based diet [91]. Specific diets in Canadian Inuit tribes [92] and Hadza hunter-gatherers [93] from Tanzania clearly showed the influence of diet on the overall microbiota composition of their feces. For the Hadza hunter-gatherers, seasonality [94] and the introduction of specific food items such as meat or honey are further hypothesized to directly change microbiota composition [95]. A meta-analysis of 27 dietary studies in human and rodents found consistent alterations of the intestinal microbiota in response to a high-fat diet and could identify a set of 228 operational taxonomic units that are able to correctly classify subjects in the dietary groups (high vs low fat diet) [96]. Adaptation of the microbiota to specific host diets is persevered across several mammalian lineages, highlighting the important role diet has on community structure [97]. Diet also had a more pronounced effect on microbiota composition compared with genetic differences in a study analysing dietary interventions in mice of different genetic background [98].

Preparation of food also plays a role on available nutrients within food, impacting microbiota composition. A cooked or raw plant-based diet led to specific microbiota changes in mice. However, this effect may specifically impact plant-based diets. The difference in microbiota changes between cooked and raw food was more pronounced for plant-based diets than for meat-based diets. A potential causal pathway explaining these differences is the digestibility and degradation of the starch as well as other plant-derived compounds [99]. Other diets composed of low carbohydrates but high-fat foods (i.e. ketogenic diet) affect microbiota changes and the immune landscape by decreasing the level of pro-inflammatory intestinal T_H_17 cells [100], showing a direct link between diet, the microbiome, and immune status. Lastly, recent research focusing on daily, longitudinal fecal sampling of 34 healthy individuals combined with detailed dietary records highlights that food choices have profound effects on the human microbiota; yet, it is individual-specific, as it strongly depends on the initial microbiota composition [101].

Urbanization has a major effect on microbial diversity and is interconnected with diet. In multiple countries around the world, an industrialized lifestyle was associated with a loss in microbial diversity compared with a more traditional lifestyle [94, 102–105], which has important implications for global health, as it is hypothesized to be a major contributing factor for the increase in non-communicable disease in the industrialized world [106]. Specifically, members of the genera *Desulfovibrio*, *Bacteroides*, *Prevotella*, *Lactobacillus*, *Treponema*, *Oxalobacter*, and lineages in the families of the *Succinivibrionaceae*, *Paraprevotellaceae*, and *Spirochaetae* have been shown to be diminished or to disappear in more industrialized contexts whereas *Akkermansia muciniphila* is more abundant [94, 103–105].

Research also postulates that diet and geography as well as lifestyle choices dictate the presence of given strains within a given species, as recently exemplified by *Prevotella copri* [107, 108], *A.muciniphila* [109], as well as *Eubacterium rectale* [110]. Studies in the USA have shown a rapid shift to an industrialized microbiota among recent immigrants and their descendants. Especially pronounced among this population is a rapid loss of taxa and encoded enzymes associated with plant-fiber degradation that increases with time spent in the industrialized world [111].

Similar patterns in microbiota changes and associated non-communicable disease have also been shown in domesticated animals, where there is a mismatch between the current living situation and the long-evolved microbial communities of their microbiota [90]. These changes in taxa, especially in the abundance of *Bifidobacterium longum* and *A.muciniphila*, have recently been shown to have a causal effect in regulating cytokine response likely through histidine and arginine metabolism [112].

Additional factors shaping the microbiota across geography include the level of sanitation in a given location, which is directly associated with exposure to pathogens. Increased exposure to pathogens affects microbiota composition through direct interaction and/or through inflammation [113]; inter-kingdom effects through non-bacterial species such as worms, protists, or fungi [114]; and exposure to drugs and antibiotics [88]. Global health disparities in infectious diseases [115], micronutrient deficiencies [116], caloric restriction [117], and undernutrition [118, 119] have all been shown to have a profound effect on the microbiome and thus on geographic differences in the microbiota profile observed.

## What is a “healthy” microbiota?

**Figure 1.**
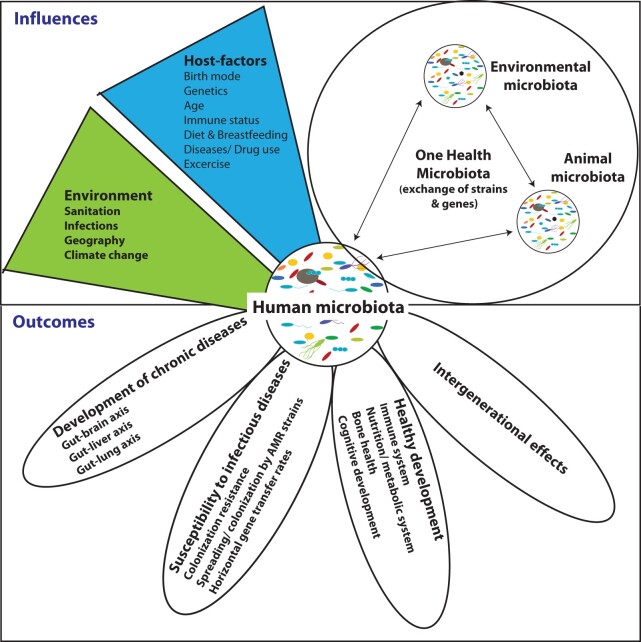
Interactions between the intestinal microbiota and global health

A healthy microbiota is neither stable in time nor the same between two individuals (reviewed in [120]). Coupled with the variety of factors that influence the microbiota, the definition of a healthy microbiota thus remains a challenge. Many of the influencing factors are tightly linked within the environment and each individual, making it challenging to disentangle specific factors influencing the growth of given members of the microbial community. In addition, the microbiota is a living entity, acquiring new microbial members as well as genetic elements through exchange of strains and genetic elements within the broader context of the environment, animals, and other humans. This concept, known as One Health microbiota, depicts the sum of genes and strains shared between humans, animals, and the environment). The combination of the human body, their larger environment, and the microbiome form creates a holobiont [121] (Figure 1).

Several “microbiota compositions” can be considered “healthy,” depending on the larger ecosystem they are part of. Likewise, the composition in given microbial strains might be stochastic whereas the overall microbial functions and the metabolic web they form seem to be more tightly linked to a general health state and more stable between individuals [10, 11].

In addition to the complex interconnectedness between the microbiota and numerous factors, the widely used amplicon sequencing approach is prone to false interpretation of healthy vs unhealthy community composition because of the strain-level differences in the microbiota. Lastly, the large geographic differences in microbiota results in various healthy microbiota communities that have been evolutionarily adapted for those populations; therefore, a universal intervention may promote a healthy microbiota in some communities while negatively impacting another community microbiota. It is critical that the microbiota is considered part of a larger holobiont that we form as a human and as a small puzzle piece of ourselves, our dietary and lifestyle choices, and the larger environment we live in. This calls for both in-depth studies and conservation of microbiota for healthy and diseased individuals, and from geographically, nutritionally, and culturally different settings.

## The role of the microbiota in the “the world’s biggest killers” of global human disease

The role of intestinal microbiota in life-course health becomes especially apparent when investigating “the world’s biggest killers,” which includes the top 10 global diseases that cause the highest number of deaths. According to the World Health Organization, “the world’s biggest killers” ordered from the most deaths to the least deaths include (i) ischemic heart disease, (ii) stroke, (iii) chronic obstructive pulmonary disease, (iv) lower respiratory infections, (v) neonatal conditions, (vi) trachea, bronchus, lung cancers, (vii) Alzheimer’s disease and other dementias, (viii) diarrheal diseases, (ix) diabetes mellitus, and (x) kidney disease [122]. The majority of these diseases have been linked to dysbiosis of the intestinal microbiota. We will provide examples of how the top three global killers are directly linked with the intestinal microbiota.

Coronary heart disease (i.e. ischemic heart disease), defined as a block to the heart blood supply, is responsible for the majority of global deaths, accounting for 16% of deaths worldwide. In the past two decades, coronary heart disease has been the most rapidly progressing type of death, rising by >2 million. In 2019 alone, it accounted for 8.9 million deaths. In patients with coronary heart disease, the intestinal microbiota has a higher alpha diversity and different microbial composition than in healthy individuals [13]. Several studies have noted that microbes used for the production of butyrate have lower abundances in the intestinal microbiota of patients with coronary heart disease [123]. It was also found that in patients with coronary heart disease, *Lactobacillales* was significantly increased whereas the phylum *Bacteroidetes* was decreased [124]. However, the causal role of the intestinal microbiota in coronary heart disease has yet to be confirmed [125].

Stroke is the second common cause of death globally, accounting for 11% of all deaths worldwide [126]. Stroke occurs when there is a reduction in blood flow to the brain, which prevents oxygen transfer and results in the death of brain cells. Perturbations in the intestinal microbiota are also found in individuals who have experienced a stroke compared with healthy controls [14]. Immediately after a stroke, researchers have found a significant decrease in the groups *Roseburia*, *Bacteroides*, and *Faecalibacterium prausnitzii* in the intestinal microbiota compared with healthy individuals [14]. The prognosis for stroke survivors was also strongly related to 18 genera that were found in the intestinal microbiota and this was corroborated in mouse models [127].

Accounting for 6% of total deaths worldwide, chronic obstructive pulmonary disease (COPD) has been shown to have distinct intestinal microbiota from healthy individuals. Compared with healthy individuals, the gut microbiome was dominated by the *Prevotella* enterotype and also had lower levels of short-chain fatty acids among patients with COPD [15]. Another study reported 146 different bacterial species from patients with COPD compared with healthy subjects [128]. Pathogenesis of the disease has also been linked to differences in metabolites, specifically choline, trimethylamine N-oxide (TMAO) and betaine, which play a role in arterial plaque formation [129]. With technological advances and a reduction in laboratory-associated costs allowing larger and especially longitudinal studies, the causal relationship between the intestinal microbiota and global diseases will become more clear [130].

## Geographic influence on gut microbial disease markers and outcomes

In the previous sections, we have shown that the intestinal microbiota is heavily shaped by geography, industrialization, and diet, and specific diseases are associated with changes in bacterial communities. However, most studies to date focusing on disease-related microbiomes are performed in industrialized countries (i.e. Europe or North America), thus leaving the question of whether the observed changes are also relevant in a global context. We briefly explore this question in the subsequent sections with two examples: (i) colorectal cancer (CRC) and (ii) childhood undernutrition.

A recent meta-analysis investigated a global signature associated with CRC across eight geographically different regions in industrialized countries [131]. The authors found that 29 species are significantly and consistently higher among CRC patients than among healthy subjects. They also found enriched protein and mucin catabolism genes, depleted carbohydrate degradation genes, and increased secondary bile-acid production in CRC patients. Similar results were obtained in an independent meta-analysis in populations of China, countries in Europe, and the USA [132], suggesting functionally and taxonomically conserved signatures for CRC, at least in industrialized countries with different dietary habits.

In the last decade, extensive research has been conducted on childhood undernutrition, including chronic undernutrition (i.e. stunting) as well as acute undernutrition (i.e. wasting). In children with acute undernutrition, a decrease in overall bacterial richness and an increase in members of the *Proteobacteria* were found compared with healthy children. Further, there was a consistent decrease in butyrate producers such as *Roseburia*, *Faecalibacterium*, *Butyrivibrio*, *Lactobacillus*, and *Bifidobacterium* (reviewed in [133]). Lastly, in severely undernourished infants, there seems to be a consistent delay in the bacterial succession observed in early life [134]. In stunted children, similar taxa are affected: there is a decrease in butyrate producers and strict anaerobes [121, 135] and an increase in pathogens/pathobionts such as *Shigella* spp. and/or *Campylobacter*. However, inconsistencies were found for alpha diversity [121, 136]. For stunting, striking similarities in the composition of small-intestinal bacteria were found in Bangladesh, the Central African Republic, and Madagascar, suggesting that the microbial composition has a direct and causal role in the disease [121, 137]. Even though studies on wasting and stunting span several countries and continents, all of the included populations consumed starch-rich food. Therefore, additional data are needed to assess signatures in nutritionally distant populations, such as pastoralists or hunter-gatherer communities.

While increasing evidence shows that specific microbial signatures are associated with global or region-specific disease, there remains a critical need for additional data to make definitive conclusions, especially in light of the technical bias found in current meta-analyses. We need larger studies assessing dysbiosis in a given disease that spans through several countries/continents and dietary habits/industrialization levels. Further, we need validation studies in experimental set-ups (i.e. animal models) in order to infer a causal relationship between the observed microbiota changes and disease outcome.

## Microbiota from a One Health perspective in global health beyond the human dimension

### Sharing of bacterial strains and pathogens between humans, animals, and the broader environment

Humans are part of a larger network comprising their direct environment as well as the animals they interact with. One Health is a concept that stresses the added value of jointly studying and addressing health problems in this interconnected space [138, 139]. The One Health concept focuses on the emergence of novel pathogens, especially among zoonotic diseases that are transmitted from animals to humans (and vice versa) as well as environmental contaminants leading to human and animal disease. With the recent increase in low-cost sequencing technology and capacity, integrative analyses that concurrently study the sharing of pathogens and commensal strains have emerged. Exchanging and acquiring microbial strains within and between animals and humans depend on exposure to a given microbial community or strain, retention of/colonization by given strains shaped through host or environmental factors, and establishment of the strain within the larger community through competition and cooperation with the larger ecosystem (reviewed in [121]).

Previous research showed that bacterial species are shared between the environment, animals, and humans. As an example, pig farms have a greater microbial diversity than suburban homes [140]. However, microbiota sharing also depends on host factors, exemplified by the higher similarities of strains shared between pig-farmers and pigs than cow-farmers and cows [141]; this sharing is mediated through indoor air [142], yet only transient [143]. Also, it has been shown that family members share the microbiota with their pet dogs, suggesting a direct spreading from non-pathogenic strains between humans and animals that are in close contact [144].

While strains sharing between humans, animals, and the larger environment can be commensal, recent evidence suggests it may cause disease. Environmental enteropathy, a chronic inflammatory disease that is linked to childhood undernutrition, is directly linked to mouthing of soil that is contaminated by chicken droppings [145, 146], proximity to animals [147], and contaminated water [147]; thus, it is favoring the spread of enteric pathogens and either symptomatic or subclinical infections [148–152]. However, evidence also shows that children living in a farm environment experience less asthma and allergy risk compared with children growing up in an urbanized environment (“hygiene hypothesis”)—a phenomenon that is likely mediated through early-life microbiota and changes induced to immune-system maturation [153–155]. There is clear evidence for the sharing of non-pathogenic microbial strains in a One Health context. However, research on the microbiome within a One Health context remains scarce. More studies are urgently needed utilizing a longitudinal design on integrated microbiota studies exploring the source, strains, direction, and magnitude of bacterial sharing.

### Microbiota as reservoirs for antimicrobial resistance

A primordial example of One Health’s relationship with the microbiota is antimicrobial resistance (AMR) [156]. AMR is conferred by specific resistance genes that are carried by bacteria. AMR is currently one of the most pressing global health problems; it is expected that multidrug resistant strains will indefinitely increase globally. Misuse and overuse of antibiotics in humans and animals are believed to be the main drivers of the emergence of resistance [156]. AMR genes are found for as long as bacteria co-exist with each other. They have spread rapidly after the broad introduction of antibiotics in medicine and agronomy. New AMR strains can be generated through gene mutations and AMR genes can be transferred from environmental strains to pathogenic/human-related strains through several mechanisms, including genetic recombination by horizontal gene transfer, conjugation, phage transduction, or transformation [157]. The human intestinal microbiota is a hotspot for AMR gene exchange due to the densely populated bacteria that are in close proximity to each other. Inter-species and intra-species competition leads to higher mutation rates, favoring the spontaneous generation of new resistance mechanisms. Further, the high cell density provides ideal conditions for exchange between transient and resident bacteria of the gastrointestinal tract.

Several other factors have also been shown to boost horizontal gene transfer between commensals and/or enteropathogens. For example, intestinal inflammation allows *Enterobacteriaceae* to lead to veritable “blooms” hence favoring gene transfer between members of this family [158]. Artificial sweeteners have also been shown to increase conjugative plasmid transfer between phylogenetically related and/or unrelated strains through activation of the SOS response and increased cell membrane permeability in the bacteria exposed to non-nutritious artificial sweeteners [159]. In line with these findings, horizontal gene transfer for intestinal bacteria was increased among people living in industrialized and urban communities compared with those living in less industrialized settings [160]. It remains to be proven if these factors thus also favor the occurence and spread of AMR genes.

AMR is found not only in humans, but in any environment where different bacteria co-exist and compete for nutrients and other resources (including animals and the broader environment). Antimicrobial carrying strains can be shared within this triangle, as can genetic material. As resistance is often conferred by mobile elements, resistance can pass between different compartments and different pathogenic and non-pathogenic strains. The widespread use of antibiotics in farm animals to increase growth results in alternative reservoirs that can harbor resistances that are then passed on to humans, potentially impacting pathogenic bacterial strains [161]. As surveillance tools for bacterial diseases, drug use, and AMR-carrying strains in livestock is still poor and undeveloped, animals present a real danger to the emergence and spread of AMR.

It is plausible that the global resistome found in human fecal samples is significantly impacted by antibiotics approved for animal use and by antibiotics used in human medicine [162]. However, there is an ongoing debate on how much resistance is shared within a given habitat (i.e. from human to human) and how much is shared between habitats (i.e. human–animal, animal–environment, or environment–human). A study in Peru showed that resistomes across different habitats are generally structured according to bacterial phylogeny and ecological gradients, yet there are given AMR genes that can cross these barriers [163]. This observation is in line with a previous study assessing >2,000 full bacterial genomes, which found that horizontal AMR and non-AMR gene transfer is mostly shaped by ecology [164]. A recent study assessing AMR transfer between farmers and their animals showed that microbial strains and AMR genes are shared more easily between farmers and pigs than farmers and other domestic animals [165]. Since the gastrointestinal tract of pigs closely resembles the gut ecosystem of humans, it is plausible that the horizontal gene transfer and AMR exchange are most likely between strains sharing the same ecology. More detailed research is needed to assess AMR exchange in the lens of One Health in order to design the best interventions to combat this global threat.

Nowadays, there is growing interconnectedness of the human, animal, and environmental habitat, exaggerated through globalization, travel, and the increasing number of persons suffering from intestinal dysbiosis. AMR and especially AMR strains in the intestinal microbiome are thus of tremendous concern for public health and could well be the next emerging pandemic we are facing.

## Microbiota-targeted interventions are promising tools to improve global health

As evidence of the importance of microbiota on health and disease accumulates, there is increased interest in intervening in the microbiota and rehabilitating dysbiotic states. These so-called “microbiota-targeted interventions/therapeutics” comprise probiotics (i.e. potential health-promoting bacteria, often isolated from fermented food), prebiotics (i.e. fibers favoring the growth of health-promoting bacteria), synbiotics (i.e. combinations of probiotics and prebiotics), and antibacterial drugs and substances (reviewed in [166–168]). Fecal microbiota transfers (FMT) have also been used especially for refractory *Clostridium difficile* infections [169, 170]. More recent interventions aim at either replacing microbial-produced metabolites (postbiotics [171, 172]) or introducing specific foods to modulate the microbiota [137, 173, 174]. Effectiveness of microbiota-targeted interventions is highly dependent on the starting microbiota of the recipient. Interventions using prebiotics require an initial presence of the bacterial group in order for it to grow (permissive microbiota). Further, microbial responses to dietary fiber are highly individualized [174]. It is unclear why interventions are highly dependent on the baseline microbiota, yet strains capable of enzymatically digesting given carbohydrates seem to play a major role [175].

For FMT, the presence or absence of given bacterial species in the donor and pre-FMT recipient microbiota can hinder or promote the succession of specific microbial groups leading from a disturbed microbial ecosystem back to a state of homeostasis. The first wave of bacteria including members of *Desulfovibrio*, *Odoribacter*, *Oscillibacter*, and *Clostridioides* genera seems to prepare the ecosystem through secretion of metabolites that helps reshape the overall ecosystem, while the second succession (including especially bacteria with bile-acid metabolizing activities) seems to lead to a restoration of “lost functions” [176]. This succession is thus favored when first-wave bacteria are present or hindered if they are absent.

**Figure 2.**
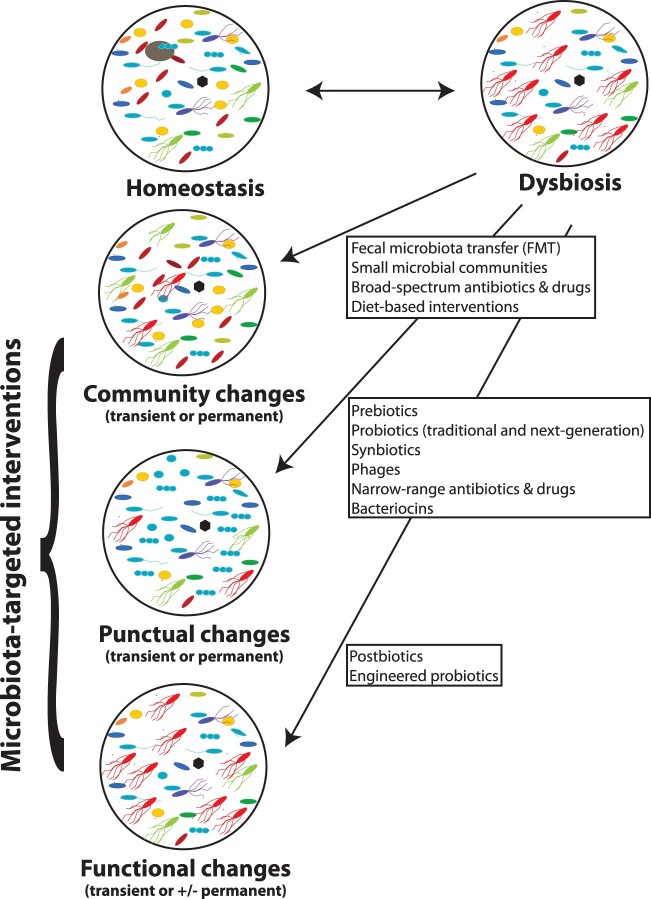
Microbiota-targeted interventions

Microbiota-targeted interventions are rapidly gaining in popularity. Current interventions aim to either (i) induce general community changes or punctual changes in health-promoting bacteria or (ii) lead to functional rather than taxonomic changes (Figure 2).

In the following section, we will discuss different interventions and their potential to curb important public health threats.

### Interventions leading to community changes

Community changes can be completed by (i) replenishing missing taxa by reintroducing complex microbial communities using fecal microbiota transplant or small synthetic microbial communities of next-generation probiotics, (ii) introducing specific dietary components favoring the growth of given groups/gilds of bacteria, such as microbiota-accessible carbohydrates (MACs) [177], or (iii) use of specific diets that are empirically tested for promoting the growth of given bacterial groups/gilds [178]. Community changes can also be mediated through broad-spectrum antibiotics and drugs suppressing a large group of bacteria (reviewed in [168]).

A first trial using an empirically pre-tested microbiota-directed food intervention has shown promising results in changing microbiota composition and ameliorating growth of moderately malnourished children in Bangladesh [137]. Clinical trials using MACs to ameliorate ill health are ongoing; however, preliminary data from mouse models indicate that MACs could have an important role in shaping the microbiota, preventing infection [179], and improving the gut–brain axis in obese mice [180]. As food is easily accessible and transportable, microbiota-directed food interventions show a high potential for improving microbiota-related detrimental health effects on a global scale.

### Interventions leading to punctual changes

Several other microbiota-targeted therapies provide punctual microbiota changes, such as pre-synbiotics and pro-synbiotics, phages, bacteriocins, and narrow-range antibiotics and drugs. Probiotics are among the longest-used microbiota-targeted interventions. More than 100 years ago, the French-Russian scientist Elie Metchnikoff hypothesized that lactic acid bacteria were able to promote longevity and have beneficial health effects by replacing “bad” bacteria such as the toxin-producing *Clostridium*. During the First World War, the German scientist Alfred Nissle further isolated an *Escherichiacoli* strain from a soldier who did not develop enterocolitis in response to shigellosis (*E. coli* strain Nissle 1917) and bacteria were used to treat gastrointestinal disease. The term “probiotic” was then coined in the 1960s by Lilly and Stillwell, who defined them as microbial-derived factors that stimulate the growth of other organisms. In the late 1980s, Roy Fuller emphasized that probiotics need to be viable and confer a positive effect on the host (reviewed in [181]).

Today, most commonly used probiotic bacteria belong to the group of *Lactobacillus* and *Bifidobacterium*. Others include the yeast *Saccharomyces cerevisiae*, some *E. coli* and *Bacillus* species, and less commonly used strains from other genera. Most of these initial probiotics were isolated from fermented food, especially milk products. However, in the last year, there has been growing interest in next-generation probiotics—a bacterial strain isolated from healthy humans. Due to the promising benefits for reducing the risk of metabolic disease, next-generation probiotics include less commonly used species (i.e. *A.muciniphila*) of live or dead bacteria. Indeed, some of these next-generation probiotics specific proteins such as isolated extract of the outer-membrane protein Amuc_1100 [182] or a secreted glucagon-like peptide [183] could ameliorate metabolic disease [184]. Butyrate-producing bacteria are a new area of interest since they are less prevalent in industrialized countries and especially in many patients with the most important public health threats including undernutrition [185], ulcerative colitis [186], and type 2 diabetes [187]. Several other bacterial strains are currently being explored as potential next-generation probiotics (reviewed in [188]). However, use of next-generation probiotics for general medical application is still a legal gray area.

Several other means of regulating the microbiota have been used or are currently being developed. The term “bacteriophages,” introduced by Felix D’Herelle in 1917, designated a hypothetical virus responsible for rapid bacterial death. Phages rapidly adopted as a means of treatment in the pre-antibiotic era have been extensively used in the Soviet medicine and have regained popularity due to the rise of AMR (reviewed in [189]). While phages are traditionally used to treat infectious diseases, their use as microbiota-modulating agents is increasingly discussed.

Last, bacteriocins may also help grow specific members of the microbiota. Bacteriocins are antimicrobial peptides that hamper growth of competing strains and are produced by specific strains of bacteria. In comparison to antibiotics, their mode of action rarely induces resistance. Further, different bacteriocins exist with either narrow or broad-spectrum killing capacities, making them attractive for biotechnological use (reviewed in [190]). More research on their mode of action as well as the spectrum of activity is needed in order to use them efficiently as microbiota-modulating drugs.

Although punctual change interventions show great potential for combating dysbiosis-related diseases in the future, their use is currently slowed down by safety issues, legal and regulatory challenges in classifying, and medical approval [185].

### Interventions leading to functional changes

Functional changes can be induced in the bacterial community either through dietary changes, specific metabolites that are ectopically administered (i.e. postbiotics), or engineered probiotics expressing given metabolites (i.e. microbiome engineering).

In previous years, microbiome engineering has been hampered due to limited availability of genetic tools to work with the gut microbiota. However, recent advances in the field of synthetic biology may help accelerate the development of strains and “smart” bacteria to help express given metabolites, thus helping to combat pathogens, diagnose early stages of cancer, regulate mood, and reduce the prevalence of metabolism or gastrointestinal disorders [191]. With the CRISPR-Cas9 technology, formerly non-engineerable bacteria such as classical or next-generation probiotic strains [192, 193] or whole microbial communities can now be genetically modified [194]. Combining the CRISPR-Cas9 methodology with phages allows single bacterial species to be genetically modified in a whole microbiome [185]. While several studies have highlighted the potential of engineered microbes, to our knowledge, no human trials have been conducted [195].

In recent years, modulating dysbiosis-associated pathophysiological changes through microbial-produced or microbial-modified diet-derived metabolites, so-called “postbiotics,” has gained popularity. Many pathophysiological changes are induced not by the bacteria per se, but by the overproduction or lack of given metabolites. Examples include the short-chain acids acetate, butyrate, propionate, and lactate that are produced by the human microbiota and have important signaling functions in the human host. Another example is tryptophan-derived metabolites (i.e. 3-indolepropionic acid), which are thought to limit intestinal inflammation by direct binding to the host receptor aryl hydrocarbon receptor (reviewed in [196]). Ectopic supplementation might provide the needed regulatory functions; however, as there are no changes to the microbiota, these metabolites have to be constantly supplied from external sources to maintain a proper signaling function. Postbiotics are now used in clinical trials to treat a variety of dysbiotic diseases.

Overall, microbiota-targeted interventions are promising tools to ameliorate and reverse dysbiosis-associated pathophysiological changes. More research is needed to understand the underlying mechanisms and evaluate the safety of these treatments for large-scale human trials.

## Conserving the microbiota for future generations

### The concepts of “missing microbes,” VANISH and BloSSUM taxa

The concept of so-called “missing microbes” [197, 198] proposes the disappearance of bacterial species, which have co-evolved with us as a human host over millions of years (so-called “indigenous microbes”). The authors speculate that this is due to our industrialized lifestyle and that their disappearance is closely linked with the rise in post-modern diseases such as asthma and obesity. The underlying reasoning is that optimal host–microbial interactions maximize the allocation and use of limited resources to benefit the host and its symbiotic microbial community. Thus, changes in the microbial ecology are having direct effects on human health, including height, weight, metabolic health, and immune development. This concept of “missing microbes” is in contrast with the earlier “hygiene theory,” which postulated a missing exposure to microbes through exaggerated hygiene [199] rather than the disappearance of given microbial taxa. As there is a vertical transmission of the microbiota from a mother to her child, the authors postulate that there is a step-wise decrease in particular bacterial species upon exposure to a more industrialized context, favoring the gradual increase in non-communicable diseases. Due to exaggerated hygiene, there is also less horizontal transfer of microbial species, accentuating the microbial decrease and leading to a complete loss of given bacterial taxa.

The concept of “missing microbes” has later been extended to the concept of “industrialization” or “Westernization” of the microbiome. Research has shown a step-wise decrease in alpha diversity (i.e. number of co-existing taxa) in the fecal microbiota from traditional hunter-gatherer communities compared with traditional but sedentary populations and industrialized countries such as the USA or countries in Europe [106]. There seems to be a consistent loss of certain taxa, termed “volatile or associated negatively with industrialized societies of humans” (VANISH) taxa. Concomitantly, we observed an increase in other taxa, termed “bloom or selected in societies of urbanization/modernization” (BloSSUM) taxa [200]. There is increasing evidence that this shift in bacterial taxa is directly associated with the rise in non-communicable diseases.

Decrease in the VANISH taxa, including species from the families *Prevotellaceae*, *Spirochaetaceae*, and *Succinivibrionaceae*, is primarily associated with a decrease in the consumption of MACs in Westernized communities [177, 201]. VANISH taxa are capable of degrading complex plant-derived carbohydrates as they encode different carbohydrate-active enzymes (CAZyme), such as glycoside hydrolase. Research shows that this microbiota transition is recapitulated by immigration of people from a country with a traditional lifestyle, such as Thailand, to a very industrialized country, such as the USA; however, this is aggravated over generations of living in the new host country [111]. Similarly, a recent experiment found that the microbiota of wild mice and domesticated mice started to resemble each other after their diets were switched (e.g. wild mice eating a domestic mice diet); it reiterates the important role of diet in global microbiome differences [90].

On the other hand, more of the BloSSUM taxa, including members of the *Bacteroidaceae*, *Enterobacteriaceae*, and *Verrucomicrobiaceae* families, were found in industrialized countries; these members are known to lead to low-grade inflammation and are favored by the highly refined, high-fat, low-fiber diet consumed in many industrialized countries [200].

Thus, a recent shift in the overall microbiota and especially a loss in health-promoting taxa seems to be associated with the rise in non-communicable diseases.

### Initiatives to conserve the world’s human microbiota

There is a rapid decline in microbial species in Westernized societies compared with traditional communities [200]. Further, non-communicable diseases are on a constant rise in Westernized societies and are likely linked to the human microbiota. This suggests that we are losing “health-promoting” bacteria and that we should conserve these taxa before they become extinct [202].

Two global initiatives have started collecting and preserving the human microbiota around the world. The Global Microbiota Conservancy focuses on isolating and conserving bacterial strains from the human fecal microbiota. The Microbiota Vault aims to conserve and characterize whole microbial communities in an international storage facility similar to the Global Seed Vault [203, 204]. While the two approaches differ in the samples they store (i.e. isolated strains vs whole microbial communities), the general idea, and the legal and ethical issues they face are similar. Further, both initiatives give the property rights of the collected strains to the local communities that provided the samples.

Besides storing microbiota for future generations, we also need to preserve our own microbiota by reducing exposure to factors that impair our microbiota. The global overuse of antibiotics, consumption of processed food and food additives, a general loss of nutritional diversity, increases in infants born by C-section, low levels of breastfeeding, and exaggerated hygiene all have their toll on the diversity of our microbiota [201]. However, this could be easily avoided. To not only maintain our microbial diversity but also sustain public health on a global scale, we should increase awareness on the important role our microbiota has in maintaining proper health and well-being.

In conclusion, the intestinal microbiota is at the cornerstone of human health and predicts the life-course trajectory for humans. Influenced by individual, environmental, and geographic factors, research on the intestinal microbiota should approach scientific hypotheses utilizing knowledge of its interplay with the larger ecosystem. Applying this approach will further our understanding on how perturbations of the intestinal microbiota impacts human health. In order to successfully change the intestinal microbiota long-term, we must have a better understanding of factors governing microbial composition and conserve the microbial diversity for future generations.

## Authors’ Contributions

Literature research: J.W., P.V. Wrote the manuscript: J.W., P.V.

## Funding

Work in PV's group is supported by an Eccellenza Professorial Fellowship of the Swiss National Science Foundation (grant number PCEFP3_194545) and the Nutricia Research Foundation (grant number 3147). This work was supported as a part of NCCR Microbiomes, a National Centre of Competence in Research, funded by the Swiss National Science Foundation (grant number 180575). PV and JW are both recipients of the excellence stipend of the Forschungsfonds of the University of Basel.
